# Supplementary material for: Three-dimensional segmentation of computed tomography data using Drishti Paint: new tools and developments
Source: R Soc Open Sci. 2020 Dec 16;7(12):201033. doi: 10.1098/rsos.201033 (PMC7813226; doi:10.1098/rsos.201033)
Supplement: A PDF contains all procedures and other supporting information for Drishti v2.7;3D surface mesh data of the segmented right cheek complex with different levels of simplification and the cropped raw data of the region of interest [file rsos201033supp1.pdf]

Supplementary materials for

3D segmentation of computed tomography data using *Drishti Paint*: new tools and developments

Yuzhi Hu[1,2], A. Limaye[3], Jing Lu[4]*

[1]Department of Applied Mathematics, Research School of Physics, Australian National University, Canberra, ACT, Australia 2601

[2]Research School of Earth Sciences, Australian National University, Canberra, ACT, Australia 2601

[3]National Computational Infrastructure, Building 143, Corner of Ward Road and Garran Road, Ward Rd, Canberra, ACT, Australia 2601

[4]Institute of Vertebrate Paleontology and Paleoanthropology, Chinese Academy of Sciences, Beijing, China 100044

*Corresponding author: lujing@ivpp.ac.cn

Table of Contents

SUPPLEMENTARY TEXT 3

Drishti v2.7 3

Installation 3

MIT Licence 3

Other resources 3

Community 3

Drishti Import v2.7 3

Drishti Paint v2.7 4

SUPPLEMENTARY TABLE 5

SUPPLEMENTARY FIGURES 1-3 6

PROCEDURES 8

1. 3D segmentation using *Drishti Paint* v2.7 8

Extracting segmented region as volumetric data 16

Extracting segmented region as surface mesh 17

2. Mesh generation in *Drishti* v2.7 19

3. Mesh simplification in *Drishti* v2.7 22

SUPPLEMENTARY TEXT

This supplementary information covers the necessary installation, user resources, essential background and supported data formats for *Drishti* (Supplementary Table 1).

Drishti has been a part of the emerging new virtual science world (Supplementary Figure 1). From 2012 to the end of 2018, some 14 scientific fields have used the *Drishti* program and benefited from the accessibility, transparency and reproducibility.

Drishti v2.7

Installation

Drishti v2.7 is available to download from https://github.com/nci/*Drishti* under "Releases".

When clicked, it will lead to a version-specific webpage where the.zip file is located at the bottom of the page; click *Drishti* v2.7.zip to download *Drishti*.

Source code and detailed information about this new release are also available on GitHub. Please note that *Drishti* v2.7 currently can run on the Windows and Linux operating systems but not MAC.

Once the download is finished, unzip the .zip file. Go to the "bins" folder, and then you should be able to run *Drishti* v2.7. *Drishti* is designed as a portable application.

MIT Licence

Copyright (c) 2011-2020 National Computational Infrastructure, Australia.

Other resources

Users can access helpful videos through the *Drishti* YouTube channel: https://www.youtube.com/channel/UCIomt3mEje4mGj_fwCNpxbQ

Tutorials and open datasets are available for practice: https://cloudstor.aarnet.edu.au/plus/s/ykqMmmikfXxHxKC?path=%2FTutorials

Community

Anyone can join the *Drishti* user community by subscribing to the *Drishti* user group via https://groups.google.com/forum/#!forum/*Drishti*-user-group/join. Besides *Drishti* user group, the authors, also help users through the social media communication channel WeChat. *Drishti* WeChat group now has more than 100 subscribers. Please feel free to email the authors to be added to both groups.

Drishti Import v2.7

Drishti Import v2.7 converts the source data into the format (. pvl.nc) which Render (8 or 16 bits per voxel) and Paint (8 or 16 bits per voxel) read.

Drishti Import v2.7 can read and convert 13 different file formats (see Figure 1 main text).

The main window (Supplementary Figure 2) is divided into two windows - the histogram window and the image window. The histogram window is used for the display of 1D histogram of the loaded volume (on the top). The image window displays the currently selected slice of volume.

***Drishti Paint* v2.7**

Drishti Paint is designed for segmentation and data cropping of volumetric data. *Drishti Paint* v2.7 (Supplementary Figure 3) allows users to segment semi-automatically/manually and generate surface mesh for regions from the volume. To facilitate the segmentation process, *Drishti Paint* v2.7 provides two modes - Graph Cut and Curves. *Drishti Paint* v2.7 has been updated and now allows input of 16 bits full resolution volumetric data.

Detail procedures of how to perform 3D segmentation using new tools in *Paint* v2.7 and generate 3D surface mesh are displayed below. [see Procedures]

SUPPLEMENTARY TABLE

Supplementary Table 1. Thirteen formats of volumetric data that can be processed by *Drishti* v2.7.

**Data format**	**Name and supporting information**
*.raw	ProRay raw triangle format;<br>a basic file format that saves the active image or stack as raw pixel data without a header
*. NIFTI	Neuroimagine Informatics Technology Initiative
TXM	Xradia X-ray transmission image data
*.jpg	Joint Photographic Experts Group
*. png	Portable Network Graphics
*.gif	Graphic Interchange Format
*.tiff/ Grayscale TIFF image	a tagged image file format
MetaImage	a particular medical image format used in the insight segmentation and registration toolkit (ITK);<br>a text-based tagged file format
Analyse 7.6 data format	Data format from software named Analyse, which is developed by the Biomedical Imaging Resource at Mayo Clinic
*.nrrd/ NRRD	a library and file format designed to support scientific visualisation and image processing involving N-dimensional raster data
*.txm/ QMUL Tom, TXM	a file format used by ZEISS Xradia 3D X-ray Microscopes
VGL	VG Studio Project file format
DICOM	Digital Imaging and Communications in Medicine<br>a standard medical imaging file format

SUPPLEMENTARY FIGURES 1-3

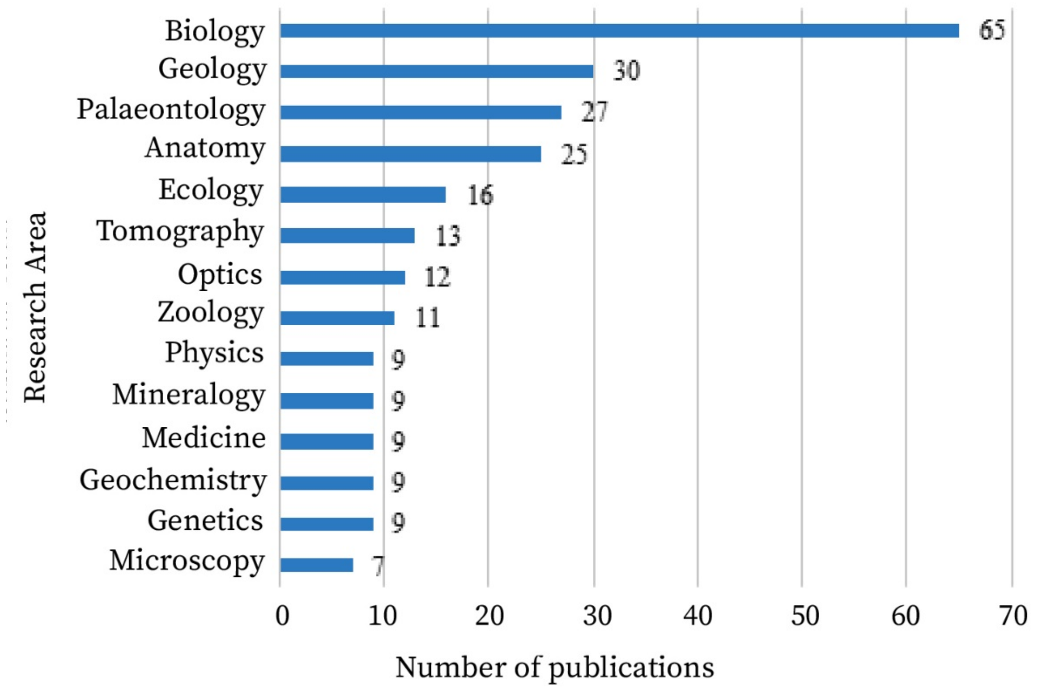

Supplementary Figure 1. Histogram of the number of publications that have used *Drishti* after Limaye (2012), [ref 24], categorised by research area. Original data from Google Scholar and Microsoft Academic. Data analysed using Microsoft Excel 2018.

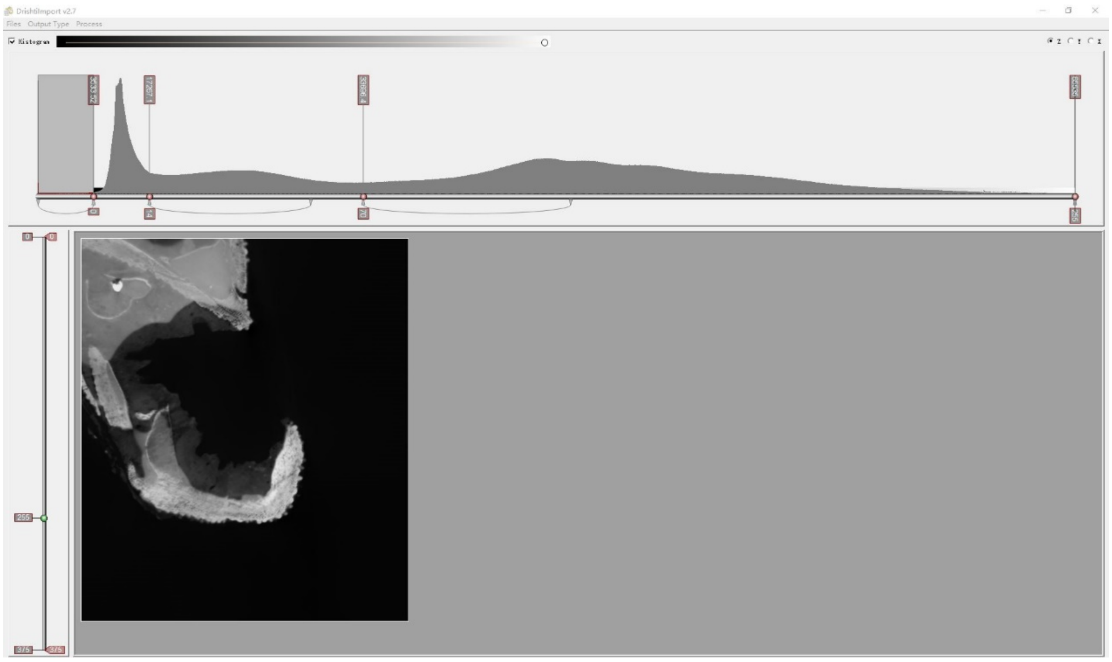

Supplementary Figure 2. A view of the main window of *Drishti Import* v2.7.

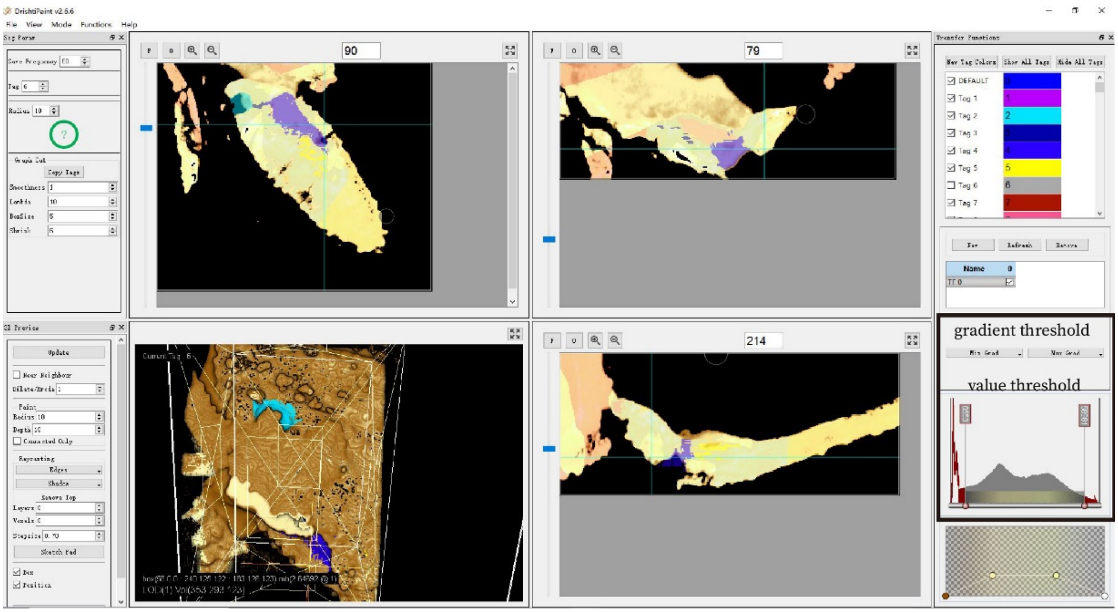

Supplementary Figure 3. A view of the user interface of *Drishti Paint* v2.7 with newly developed value and gradient multi-thresholding for both Graph Cut and Curves modes highlighted using a black box.

PROCEDURES

1. 3D segmentation using *Drishti Paint* v2.7

Download the provided volume data from Fig share:
V244_2015_selected-16bits.pvl.nc and V244_2015_selected-16bits.pvl.nc.001

Once the download is completed, open *Drishti Paint* v2.7, to show the following screen.

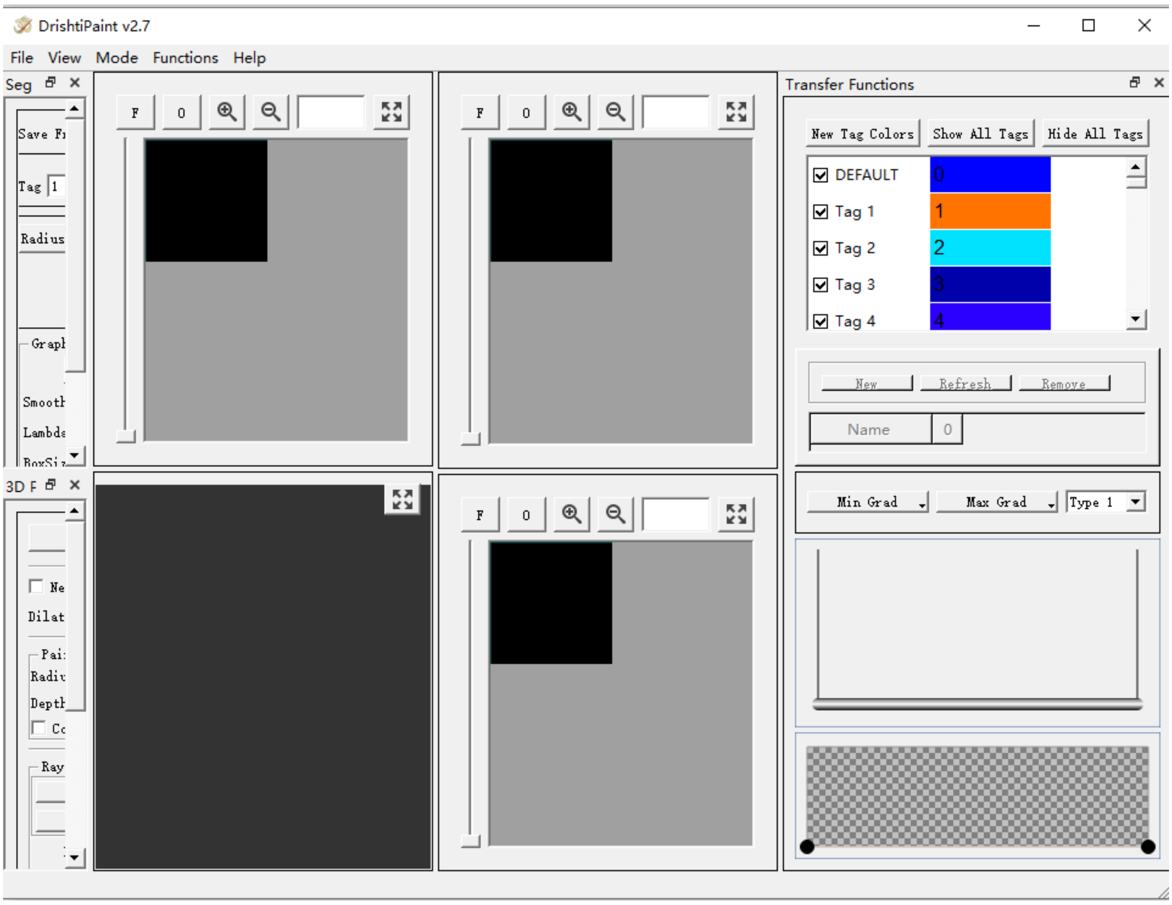

Import V244_2015_selected-16bits.pvl.nc by dragging it into *Drishti* Paint or clicking "File" →"Load"→ select V244_2015_selected-16bits.pvl.nc.

Select "1" when the Subsampling Level dialogue pops up.

[**Note** You can also subsample the volume data to ensure a quicker response while segmenting it if you do not have a good graphics card. Subsampling level 1 means keep the data as it is.]

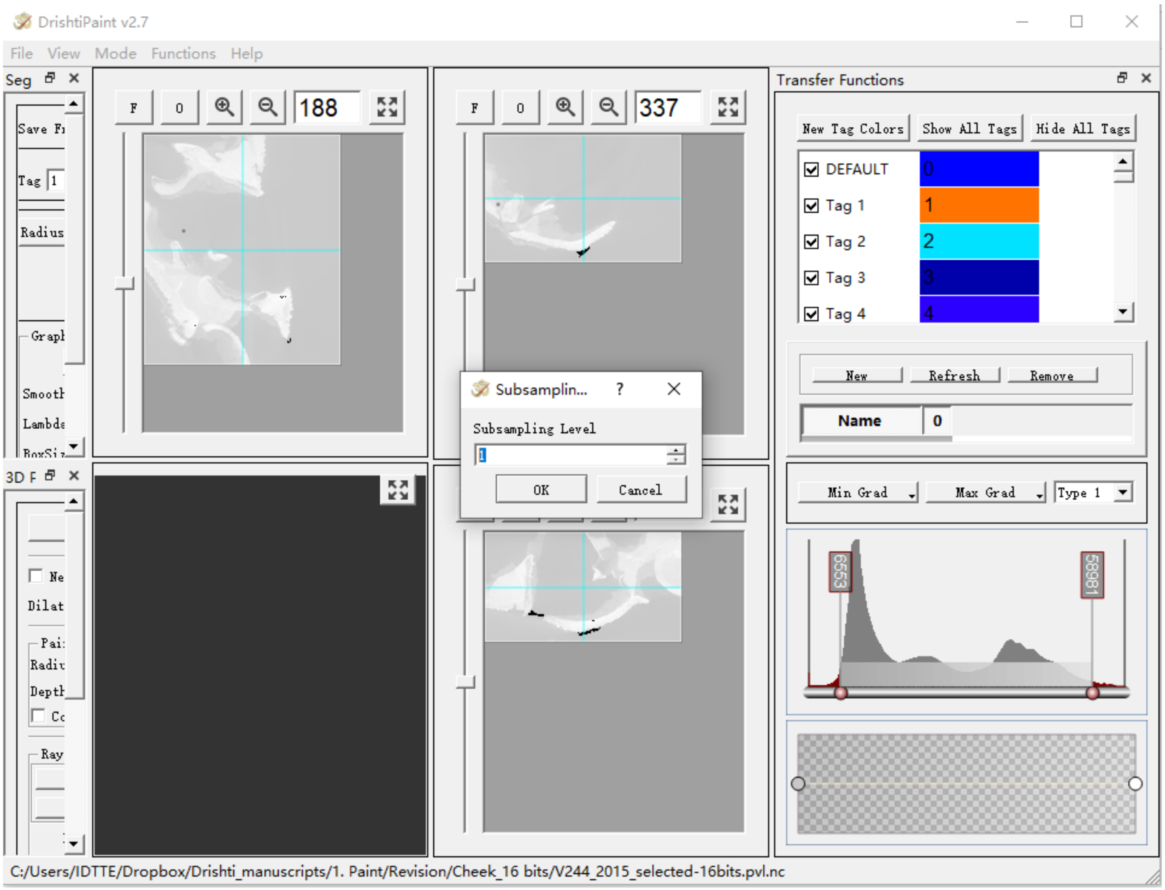

There are two modes in Paint v2.7, and the default mode is "Graph cut" which we use to segment the right cheek complex, as shown below.

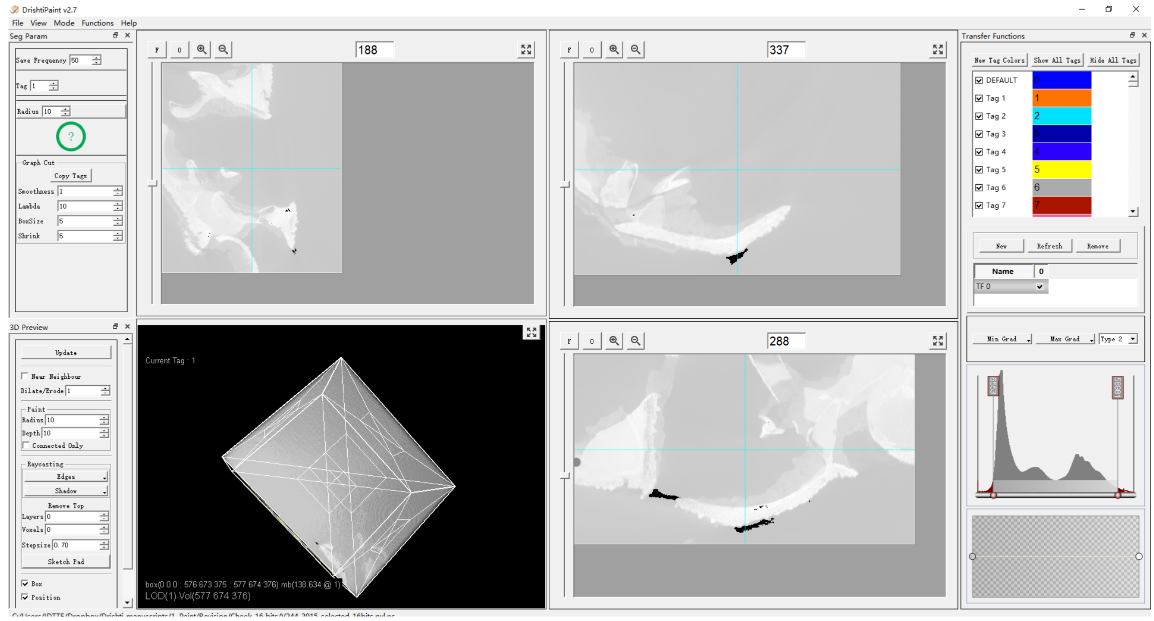

Change the colour and opacity of the data by hovering over the Colour-Opacity Gradient Interface, then press the space bar.

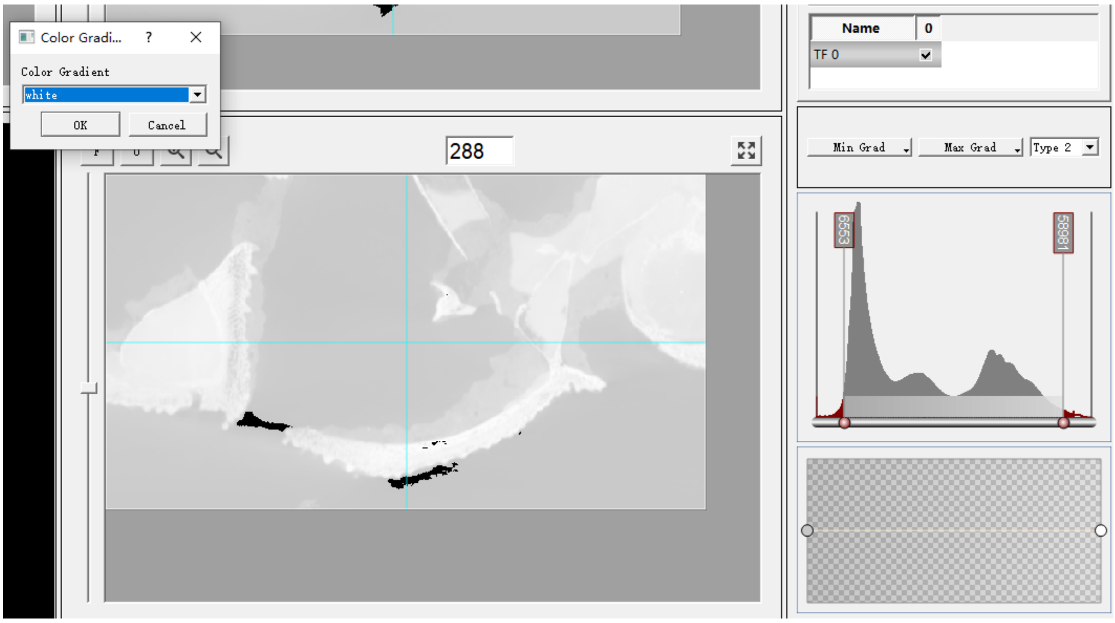

Change the Colour Gradient from white to cream-brown. [Feel free to change to any colours you prefer.] Then adjust the Transfer function Interface until the right cheek complex is shown clearly in the 3D view window at the bottom right of the four split screens, as below.

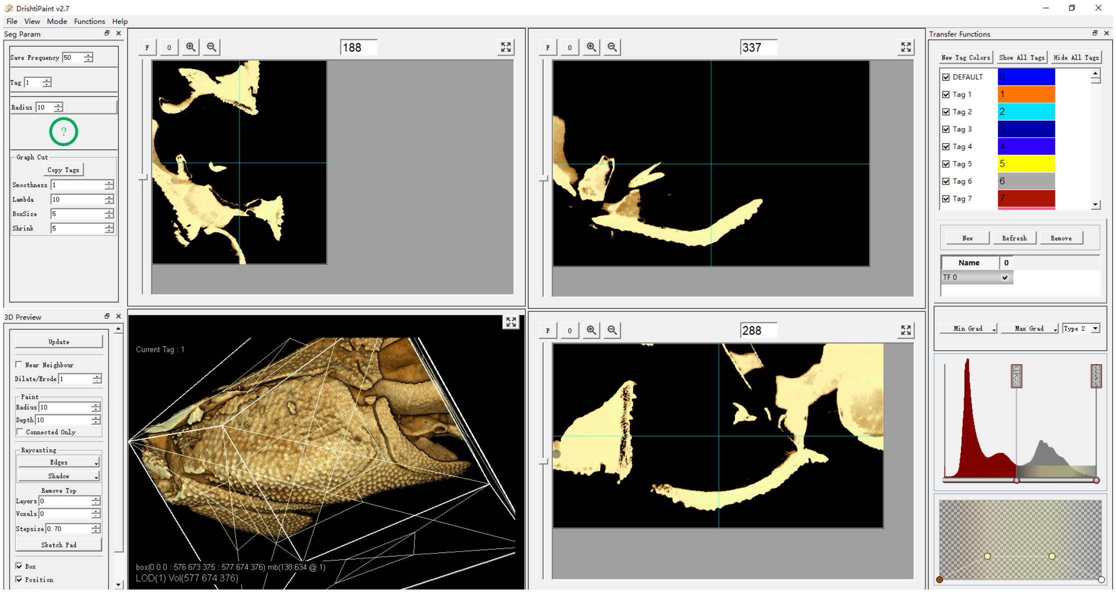

Adjust the cream-brown gradient to clear the volume, as shown.

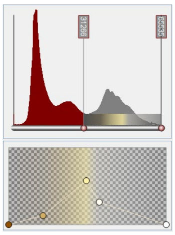

A cleared volume displays as below.

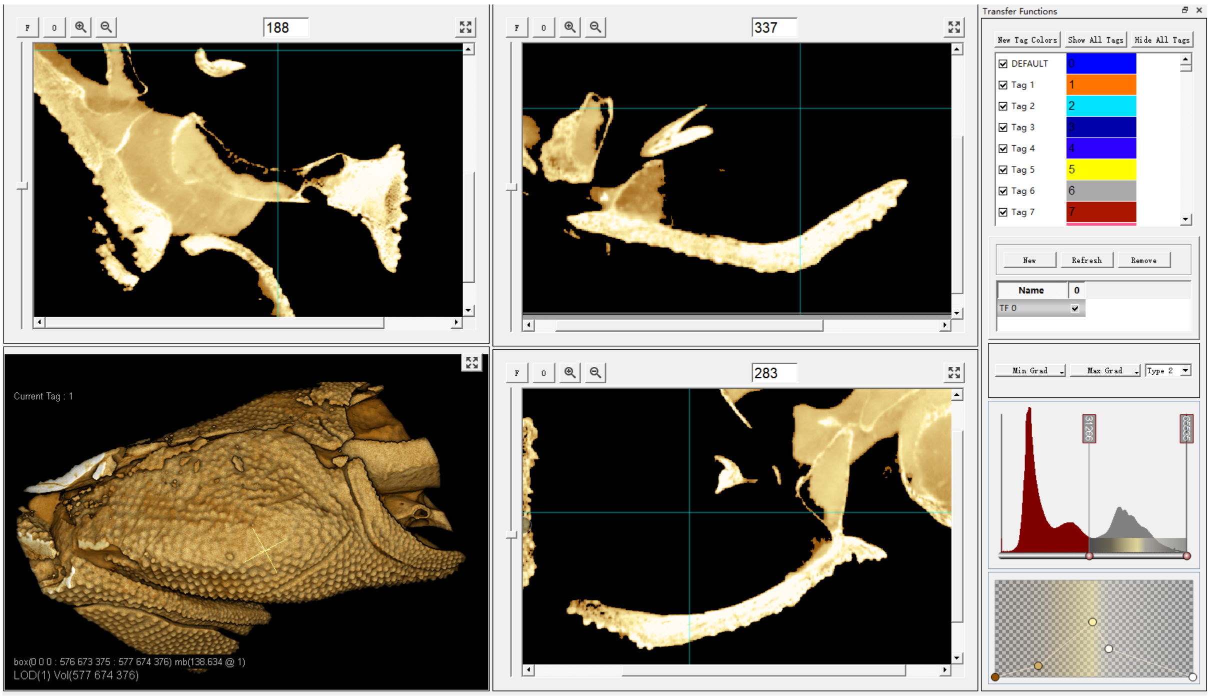

Look at this data firstly by:

1. Using arrow keys/mouse wheel or the slider bar on the left to move between slices.
2. Using Up/Down arrows to move to next/previous slice or Mouse wheel to move to next/previous slice.

Adjust the image size by using the F, O, + and - buttons for changing the image size.

- O: Original image size.
- F: Fit image size to the current window size.
- +: Increase image size.
- -: Decrease image size.

On the left panel, there are two separate panels- Seg Param and 3D Preview. Both panels are free to move anywhere on the screen. On the Seg Param panel, shown below, one can choose any tag number. Select tag number from the Tag box.

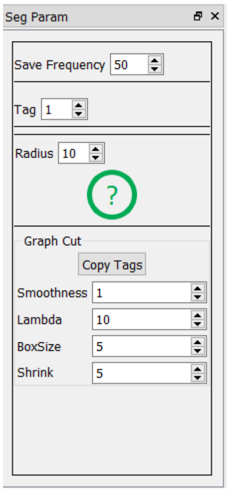

Change tag colour by clicking on tag colour patch in the "Tag Colour Editor". Double click the tag colour patch to change the tag colour.

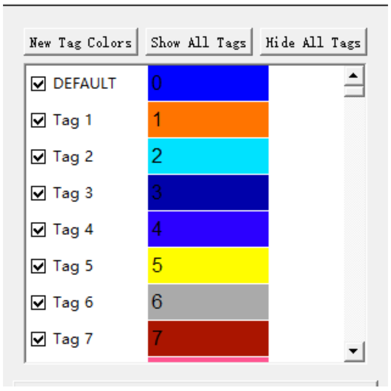

Under the "Graph Cut" mode, The Paint dot can be changed by adjusting the Radius and Depth for a better tagging result. Every dataset is different, so one will need to take time to find out the best setting. Tagging result also depends on the Smoothness, Lambda, BoxSize and Shrink features.

- Smoothness: can be used for smoothing, dilation and erosion operations.
- Lambda: controls the tightness of the curvature object region as defined by the graph cut algorithm. It is internally used to increase/decrease the average gradient magnitude
- BoxSize: indicates the average gradient magnitude which is calculated over the region of size.
- Shrink: can be used when copying tag from the previous slice to be used as seeds for tagging operation for the next slice.

The YouTube channel contains general tutorials for how to perform 2D Graph Cut, 3D Painter. We suggest using those tutorials for a detailed guide for short cuts and general introduction.

Here, we demonstrate new tools in Paint v2.7- gradient thresholding with 3D Free Form Painter.

Use gradient thresholding to clean this data further and remove unwanted noise, for example, matrices inside the fossil during preservation.

For this particular data, we used the Type 2 gradient thresholding.

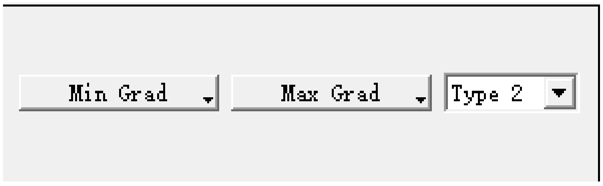

The Min Grad and Max Grad can be adjusted whenever needed while using 3D Freeform Painter to segment this data.

Use 100 Min Grad and 100 Max Grad to identify the boundaries between the right cheek complex and other unwanted elements in this data.

Then tick both Near Neighbour and Connected Only boxes to ensure your 3D segmentation only considers the connected regions.

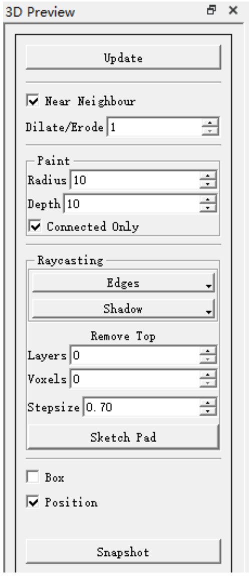

Use tag 1 to segment the cheek complex. Hold Shift then use the left mouse button to paint in the 3D view window. We used Radius 30 and Depth 12.

[Note: you can use any value as long as it gives you the correct depth and includes your region of interest.]

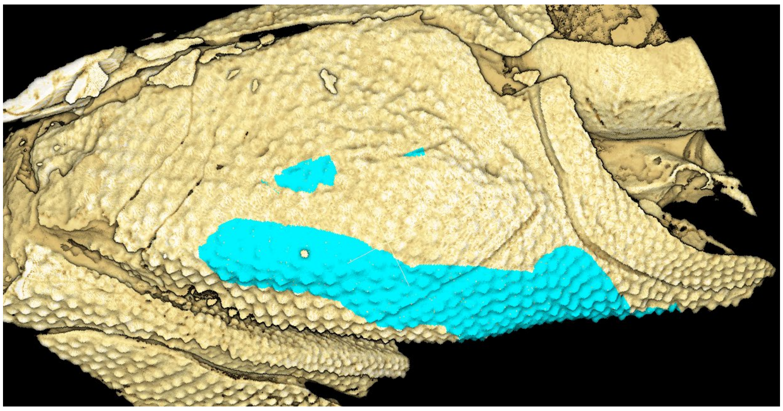

Zone into the tagged region and identify the small bits missed, for example:

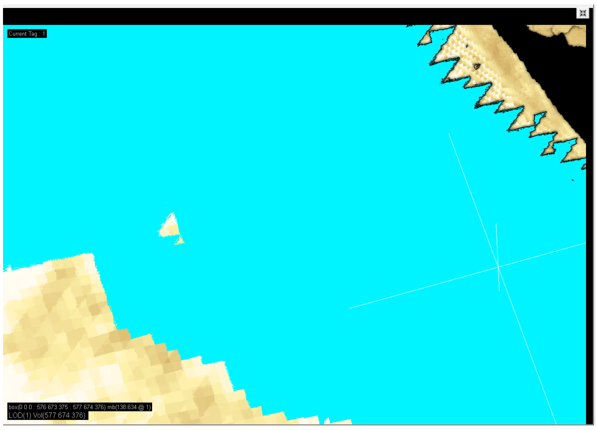

Fix this by pressing "D" to perform a dilation.

[**Note** When dilating the tagged region of interest, a pop-up message may appear- "cannot dilate. You are on voxel with tag 0, was expecting tag 1". This occurs only when the cursor is not on a voxel with the appropriate tag value. The Near Neighbour switch under 3D Preview parameter panel gives a better view of voxels (default is trilinear interpolation of voxels which gives smoother rendering but may obstruct appropriate voxel selection). This helps to select the correct voxel.]

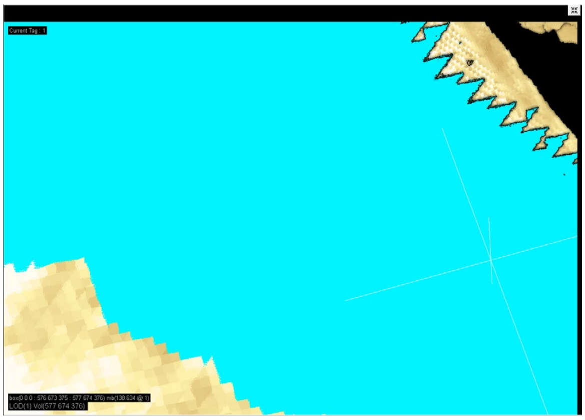

Repeat when all spaces have been filled using tag 1. Then Continue.

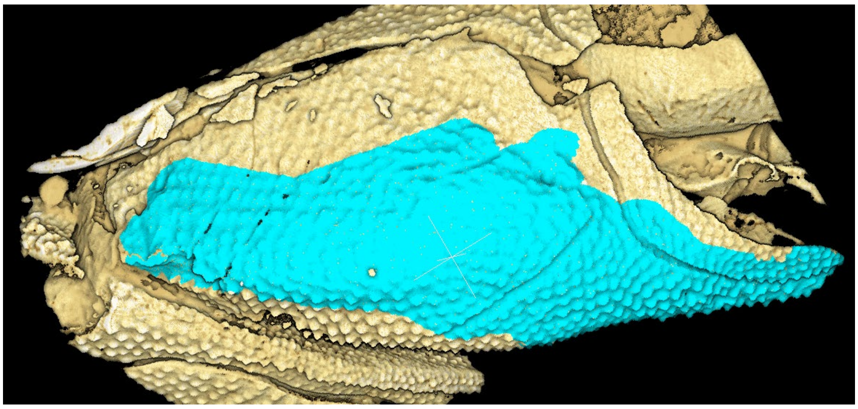

Adjust Min and Max Grad, Radius and Depth for different regions on the cheek complex, then paint the whole element. Unclick tag0 to hide all unwanted regions and only display tag 1, as below.

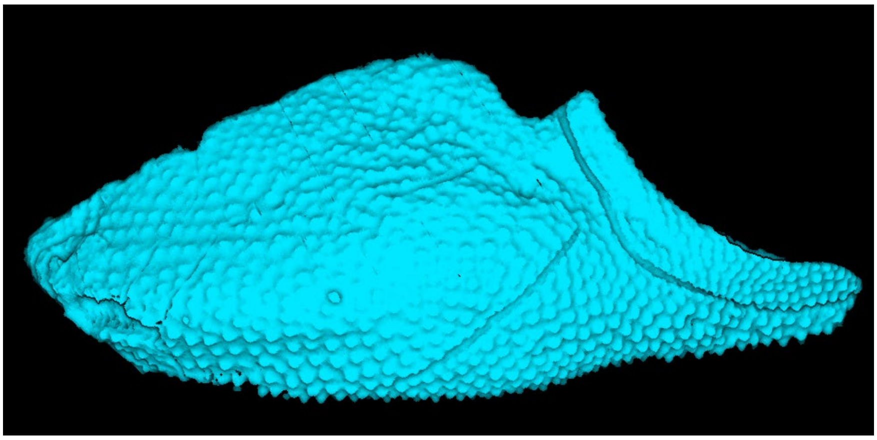

The segmented cheek complex can be extracted as either as volumetric data or surface mesh.

Extracting segmented region as volumetric data

To extract segmented volume, go to File then Extract Tagged Region. Then select 1 for tag1. Click Ok to proceed.

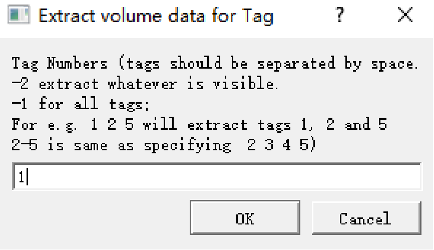

Choose Tag only.

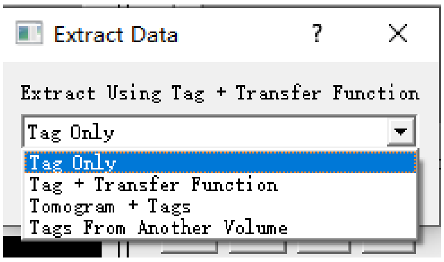

Set Outside value as 0, then click Ok to proceed.

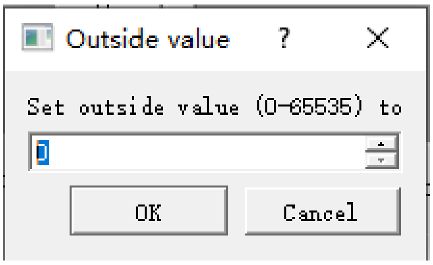

Name the file.

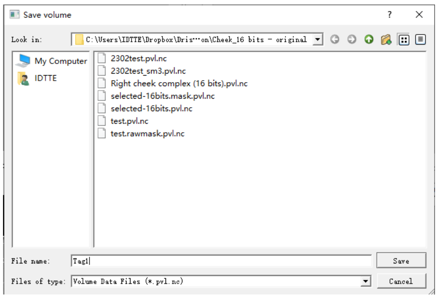

The segmented volume data of this segmented right cheek complex is now saved to the host folder- Tag1.pvl.nc & Tag1.pcl.nc.001. The saved volume can then be imported in *Drishti* or any preferred software.

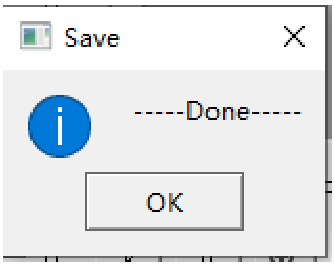

Extracting segmented region as surface mesh

To extract segmented volume as surface mesh, go to File then Mesh Tagged Region. Then select "1" for tag1.

Click Ok to proceed.

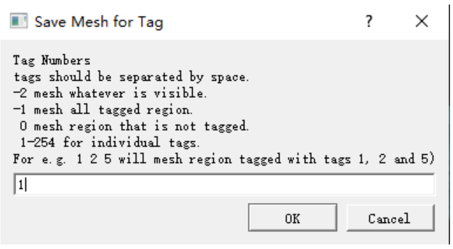

Choose the colour for this mesh using the drop-downs:

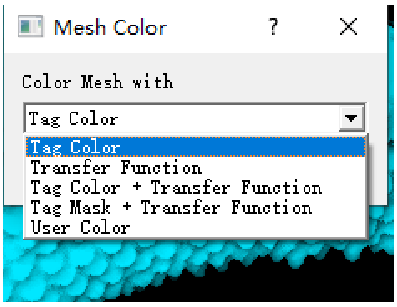

Choose No Scaling or Yes depends on what do you want to do as the next steps. Click Ok to proceed.

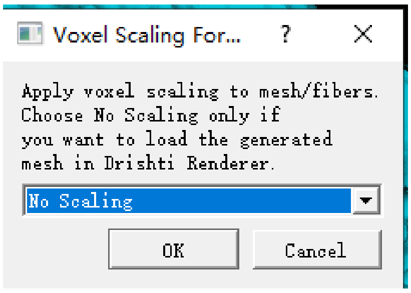

Enter "1" if you do not want any subsampling. You can also enter any number if you want to subsample your surface mesh. Click Ok to proceed.

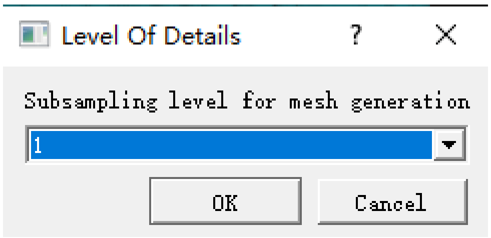

Enter the file name to save this surface mesh

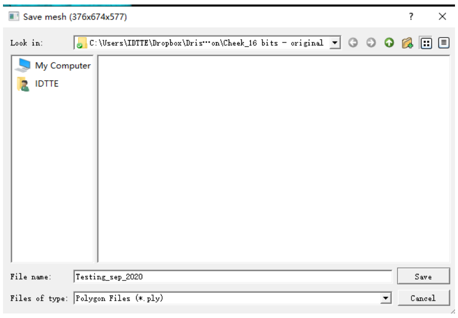

You can choose to close holes for this surface mesh data. In this case, we did not want to close any holes as we want to keep the original surfaces.

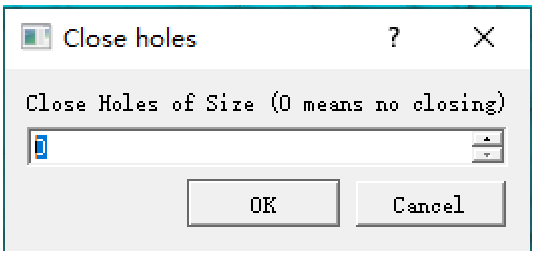

You can choose to smooth or not smooth your volume data before Paint meshing the surfaces. Here, we used the smooth factor of 3 as an example. Feel free to use any smooth factor until you get the ideal results you prefer for your data.

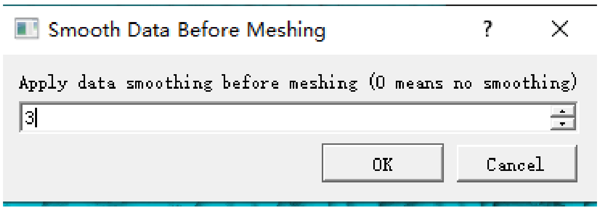

You can also choose to smooth your mesh. We used a factor of 3 as an example.

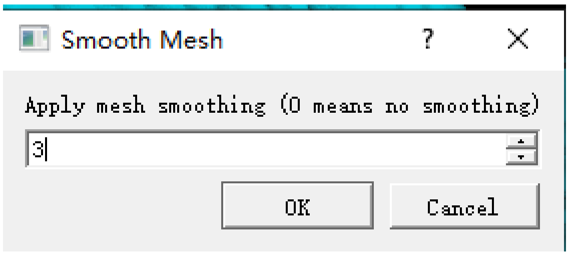

You should now have the surface mesh data of this segmented right cheek complex saved to your preferred folder. If you have followed all the steps identically, you can compare your saved output with the provided file- Testing_sep_2020.ply in Meshlab or any preferred software.

2. Mesh generation in *Drishti* v2.7

Import Tag1.pvl.nc into Drishti v2.7. By default, Drishti is in Low-resolution mode, as shown below.

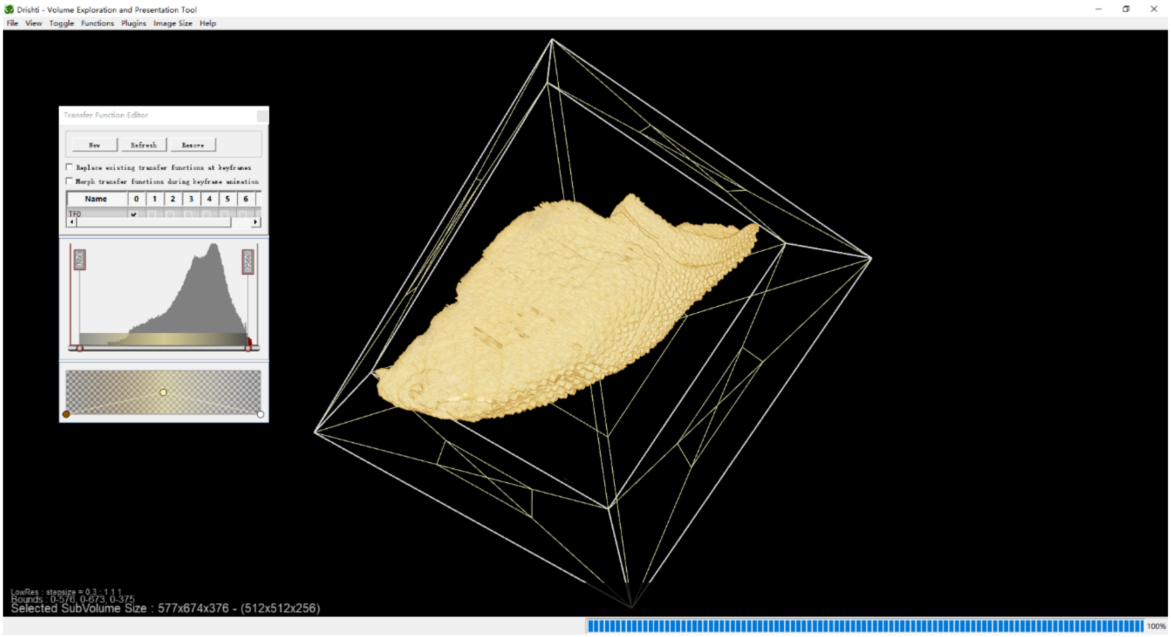

Press F2 to bring up the High-resolution mode. Select the transfer function that represents the dataset. Then adjust the Colour-Opacity Gradient Interface to "cream-brown" colours then adjust the opacity to observe this data.

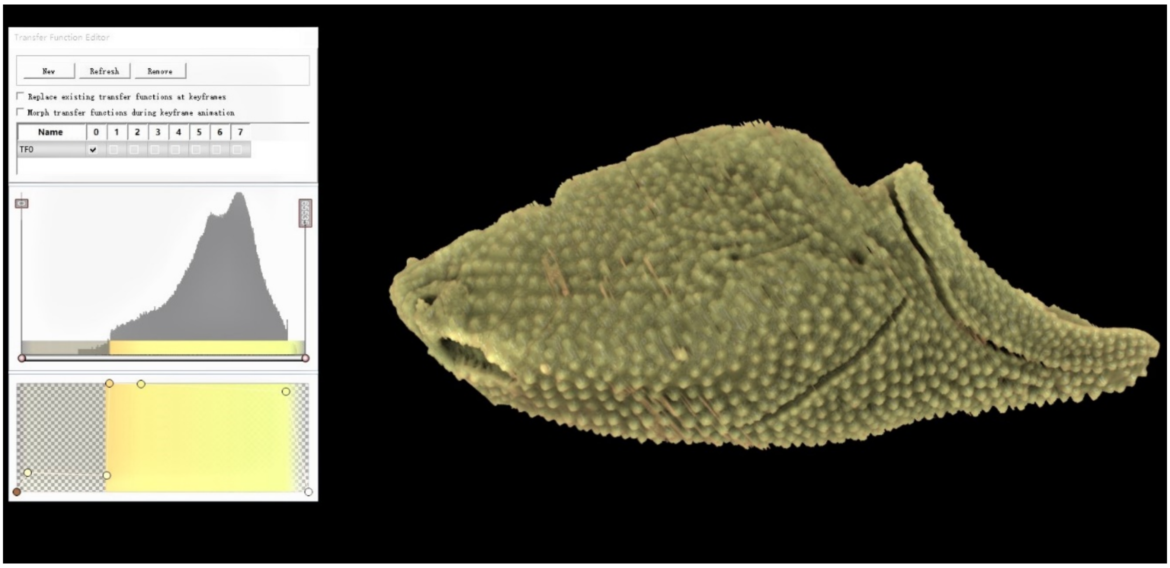

Click on Plugins on the toolbar locates at the top right corner of the screen.

Select Mesh Generator from the dropdowns. Then you should be able to see the pop-up window for Mesh Generator Parameters.

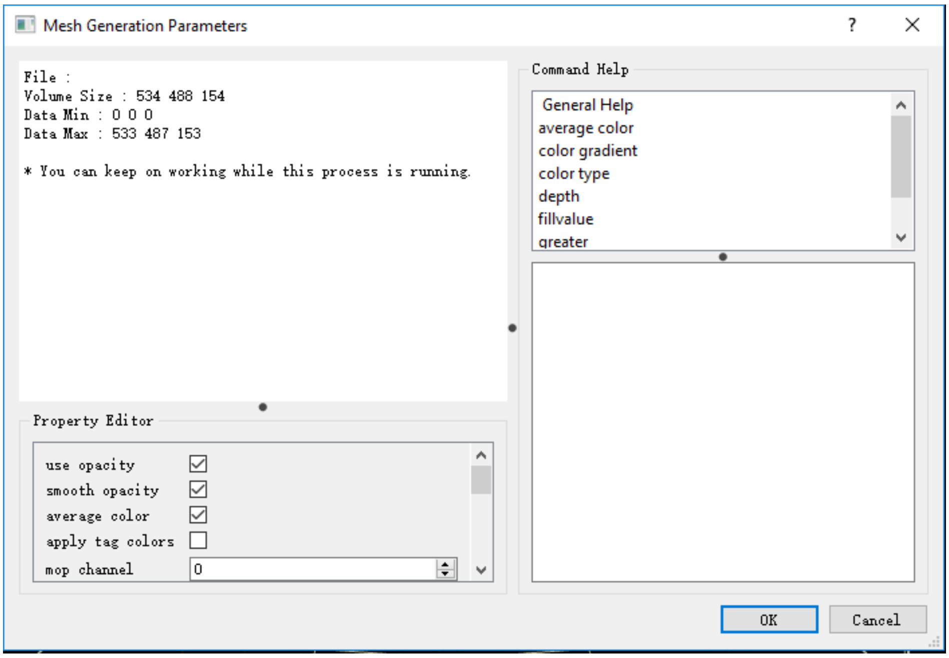

Under Property Editor, Depth is the parameter that is very useful for palaeontological data. Depth gives the user a possibility to texture the surface with the inside structure - it reveals what is "beneath the skin". On the other hand, adjusting Depth can help to make a non-distinct feature look clearer on the surface of the object.

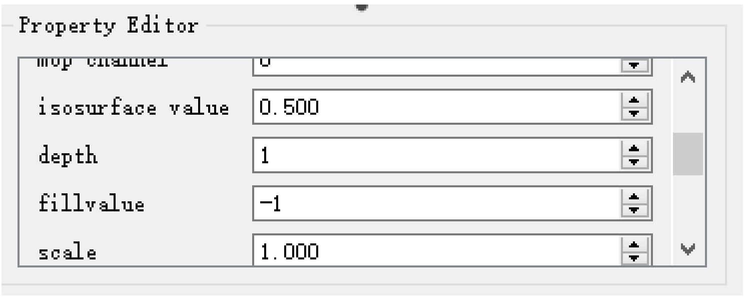

Type 3 for depth. Then press Ok to continue.

[**<u>Note</u>** User can specify how much memory is used for mesh generation. If not enough memory is available to accommodate all the required data, then the mesh will be generated in several slabs. These slabs are then joined together to form the complete mesh. We usually put 4 GB for a larger dataset (9GB), however how much memory you want to use is truly depending on your hardware and settings.]

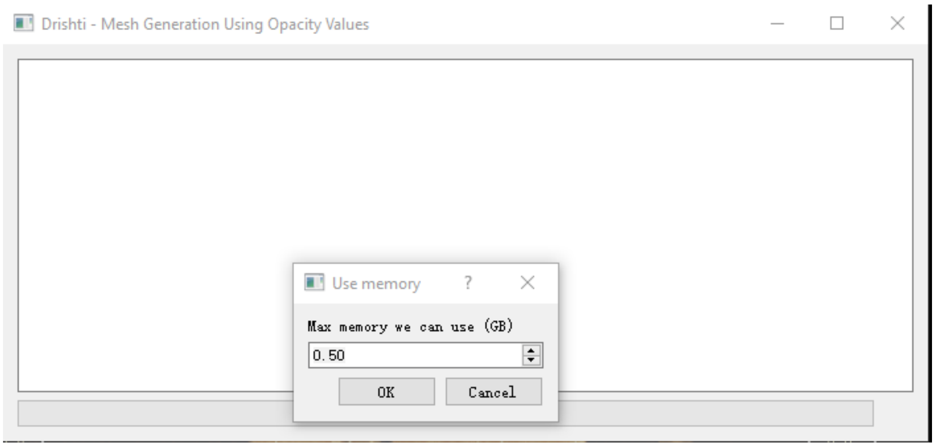

Click Ok to continue.

A pop-up window will appear to ask where you want your mesh to be saved. Select a preferred location then click Save.

If you follow all the steps above, your output should be the same as the provided data file: Cheek_Mesh.ply

Please note that both *.ply and *.stl formats are available when exporting mesh.

Your saved mesh will then be ready to import into your preferred 3D printing software.

3. Mesh simplification in *Drishti* v2.7

Import Cheek_Mesh.ply into *Drishti* v2.7
or
Click on Plugins on the toolbar locates at the top right corner of the screen.

Then select Mesh Simplify from the dropdowns. Then *Drishti* will ask you to select a mesh data to import. Select Cheek_Mesh.ply.

Drishti then asks what name and where do you want to save this simplified mesh data, enter the preferred name and pick a location to save this file. Once finished, the Mesh Simplification Parameters dialogue will pop up:

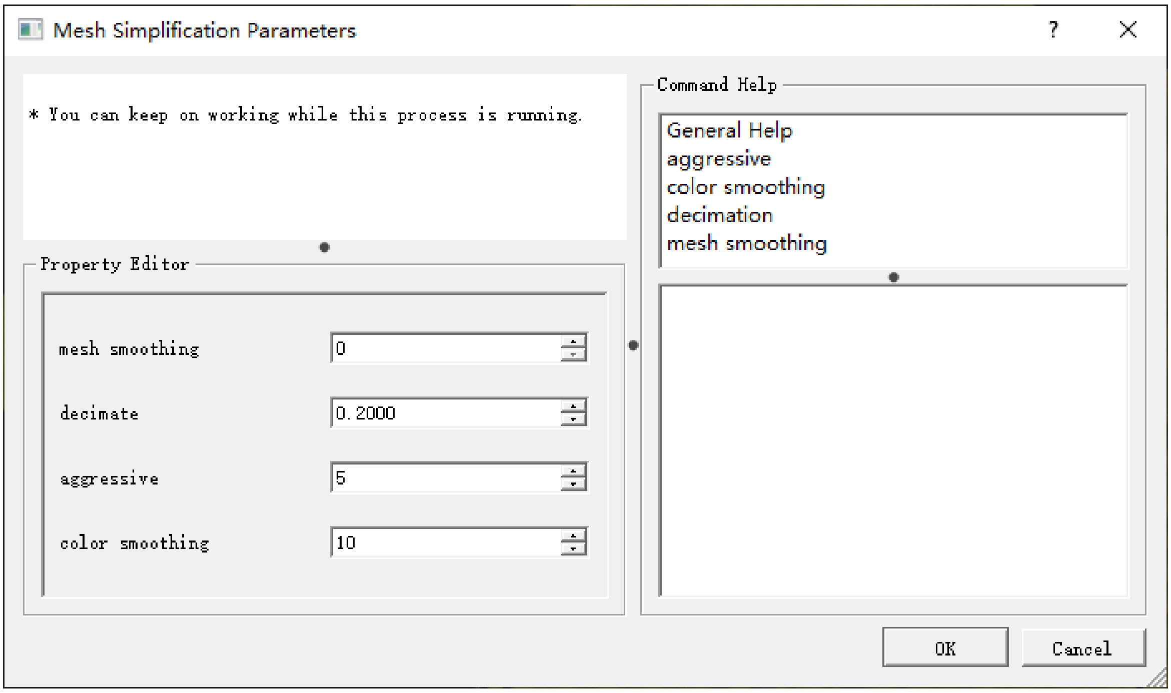

Feel free to play with the four properties. Under the Command Help, we also listed all the information/explanation of each property.

Let use: mesh smoothing 2 and decimate 0.5 (50%) as an example. Click Ok to perform this mesh simplification.

The simplified mesh is now saved: Cheek_Mesh_Simp_test.ply.
